# Grape Seed Proanthocyanidins Improve White Adipose Tissue Expansion during Diet-Induced Obesity Development in Rats

**DOI:** 10.3390/ijms19092632

**Published:** 2018-09-05

**Authors:** Aïda Pascual-Serrano, Cinta Bladé, Manuel Suárez, Anna Arola-Arnal

**Affiliations:** Nutrigenomics Research Group, Departament de Bioquímica i Biotecnologia, Universitat Rovira i Virgili, 43007 Tarragona, Spain; aidasbk@gmail.com (A.P.-S.); manuel.suarez@urv.cat (M.S.); anna.arola@urv.cat (A.A.-A.)

**Keywords:** adipocyte, adipogenesis, flavonoids, hyperplasia, hypertrophy, polyphenol

## Abstract

The development of metabolic complications associated with obesity has been correlated with a failure of white adipose tissue (WAT) to expand. Our group has previously reported that a 12-week administration of grape seed proanthocyanidin extract (GSPE) together with an obesogenic diet mitigated the development of cardiometabolic complications in rats. Using the same cohort of animals, we aim to elucidate whether the prevention of cardiometabolic complications by proanthocyanidins is produced by a healthier expansion of visceral WAT and/or an induction of the browning of WAT. For this, adipocyte size and number in retroperitoneal WAT (rWAT) were determined by histological analyses, and the gene expression levels of markers of adipogenesis, browning, and WAT functionality were quantified by RT-qPCR. The long-term administration of GSPE together with an obesogenic diet expanded rWAT via an increase in the adipocyte number and a preventive decrease in the adipocyte size in a dose-dependent manner. At the molecular level, GSPE seems to induce WAT adipogenesis through the upregulation of peroxisome proliferator-activated receptor (Pparγ) in a Sirtuin 1 (Sirt1)-dependent manner. In conclusion, the healthier visceral WAT expansion induced by proanthocyanidins supplementation may explain the improvement in the cardiometabolic risks associated with obesogenic diets.

## 1. Introduction

In obesity, fat is excessively accumulated in white adipose tissue (WAT) under conditions of energy surplus or reduced energy expenditure. The prevalence of obesity is increasing, and this is dangerous, since the risk for cardiovascular complications and type 2 diabetes among other diseases increases with obesity [1]. However, it seems that the total fat mass may not be the direct link between obesity and its associated diseases, since some obese individuals are relatively metabolically healthy [2]. In fact, the capacity of the WAT to expand to store the excess energy may prevent the development of metabolic complications in response to the surplus energy, since it may prevent the lipids from targeting other organs [3]. However, WAT can only expand up to a certain threshold, beyond which their capacity to store more fat is diminished, which is also associated with metabolic complications [4]. In this regard, several of the obesity-related metabolic complications might be explained by WAT dysfunction as a consequence of an impaired capacity of the fat depots to store more energy [5].

With a continuous energy surplus, the expansion of WAT occurs first via the increase in adipocyte size (hypertrophy) and then via the increase in adipocyte number (hyperplasia) and/or hypertrophy [4]. Thus, adipocyte hypertrophy has been linked to an increased risk of developing metabolic diseases related to obesity, whereas a protective effect has been attributed to adipocyte hyperplasia against obesity-related metabolic alterations [5]. Hyperplasia is produced by the differentiation of preadipocytes (i.e., adipogenesis), and a cascade of transcription factors controls this process. Among these transcription factors, peroxisome proliferator-activated receptor (Pparγ) is considered the master regulator of adipogenesis, and coregulators such as the Pparγ coactivator-1 alpha (Pgc1α) can in turn modulate its activity [6]. In fact, Pparγ controls the functionality of WAT by regulating not only WAT lipogenesis, but also the browning of WAT [7].

Brown adipose tissue (BAT) is characterized by a large number of mitochondria and thermogenic capacity, and thus the remodeling from WAT to BAT, which is known as the browning process, also has beneficial effects on obesity-related disorders [8]. BAT uses the uncoupling protein 1 (UCP1) for its thermogenic function, and UCP1 is the main marker of brown adipocytes. Among others, the expression of the Ucp1 gene is controlled by Pparγ, Pgc1α, and the PR domain containing 16 (Prdm16).

Proanthocyanidins are a class of flavonoid compounds that are present in many food and drinks such as fruits, vegetables, and red wine, and thus they are very abundant in the human diet [9]. In particular, grape seeds are rich in this class of flavonoids. In fact, various studies using a grape seed proanthocyanidin extract (GSPE) have demonstrated its capacity to improve metabolic complications associated with obesity [9], including its ability to reduce blood pressure [10], act as a hypolipidemic agent [11], and improve inflammation [12] and insulin resistance. Specifically, previous studies from our research group provided evidence showing that the daily administration of GSPE (at doses of 25 mg/kg per day, 100 mg/kg per day, and 200 mg/kg per day) for 12 weeks together with a diet high in carbohydrates and fat (i.e., cafeteria diet or CAF diet) in rats can mitigate the increase in blood pressure and plasma lipids levels in response to the obesogenic diet. However, only with the dose of 200 mg GSPE/kg body weight (BW) was a slight attenuation of the CAF diet-induced increase in BW observed [13]. Therefore, by using the same cohort of animals, the objective of this study was to elucidate whether the GSPE-mediated attenuation of the development of obesity-associated cardiometabolic risk factors due to the prolonged feeding of the CAF diet is due to a healthier expansion of visceral WAT and/or the induction of the browning process of the visceral WAT.

## 2. Results

### 2.1. Daily Administration of GSPE with a CAF Diet Reduces Plasma TG Levels without Affecting Adiposity

In this study, we used the same cohort of animals as the previous study reported by Pons et al. [13]. That study reported that the daily administration of GSPE with the CAF diet did not prevent BW gain during the 12 weeks of the experiment, although with 200 mg GSPE/kg BW, a tendency to avoid the obesogenic diet-induced increase in BW was observed by the end of the experiment [13]. Similarly, the daily administration of GSPE with the CAF diet did not alter the caloric intake nor prevent the alteration of the adiposity index of the animals compared with the CAF diet-fed rats without GSPE (Table S1). However, the CAF diet-induced retroperitoneal WAT (rWAT) accretion was slightly attenuated by the administration of 200 mg GSPE/kg BW per day. Moreover, a similar effect was seen in the weight of subcutaneous WAT (sWAT) with 100 mg GSPE/kg BW.

Although the daily administration of GSPE together with the CAF diet for 12 weeks did not improve the adiposity index, it improved the plasmatic lipid profile, as previously reported by Pons et al. [13] in the same cohort of animals. Specifically, the plasmatic TG levels of the rats fed a CAF diet for 12 weeks were higher compared to the animals fed the standard (STD) diet, and when the obesogenic diet was supplemented with the daily administration of 25 mg GSPE/kg BW or 200 mg GSPE/kg BW, the plasma triglyceride (TG) levels decreased by 28% or 25%, respectively (Table 1). Moreover, the plasmatic glucose (GLU) and leptin levels were also increased in the animals after 12 weeks of CAF diet compared to the STD diet-fed rats, but the daily administration of GSPE with the CAF diet did not change these parameters (Table 1).

### 2.2. Daily Administration of GSPE with a CAF Diet Prevents the Failure of the rWAT to Expand

**Figure 1:**
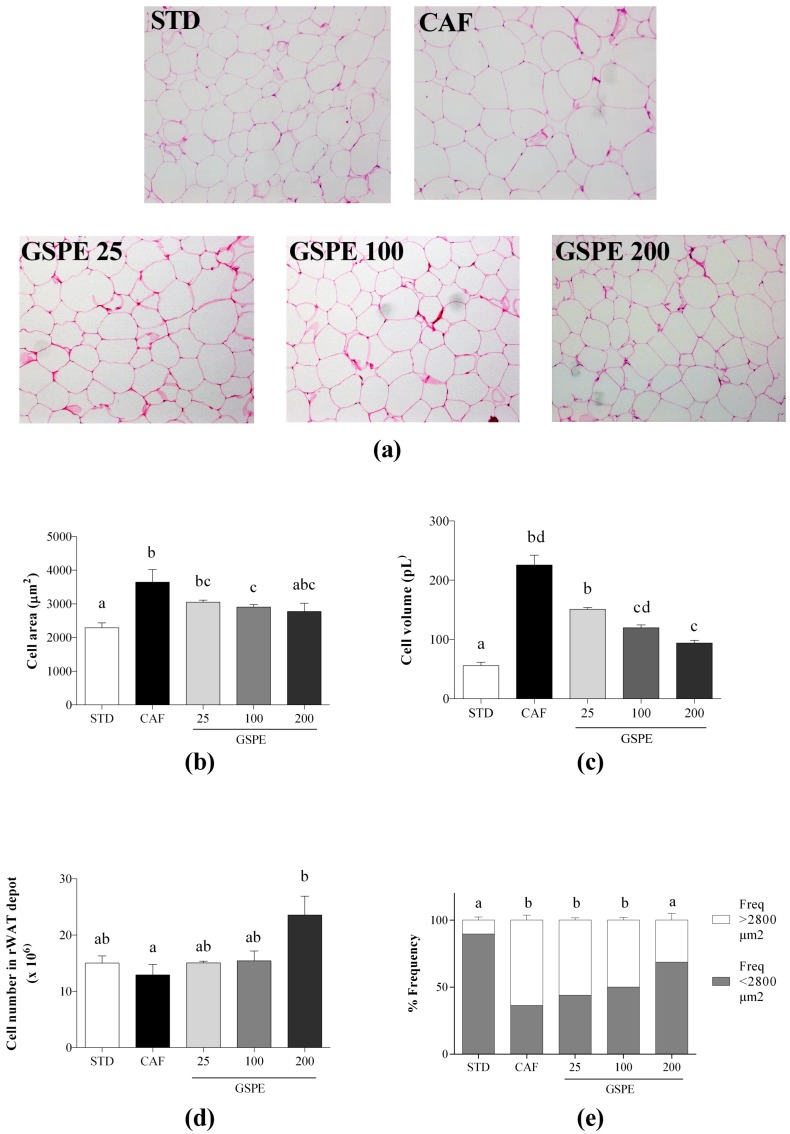
Effect of the long-term administration of a grape seed proanthocyanidin extract (GSPE) with an obesogenic diet on the size and number of visceral adipocytes. Rats were fed either a standard chow diet (STD group, n = 10), a cafeteria diet (CAF group, n = 10), or a cafeteria diet with daily administration of 25 mg GSPE/kg BW, 100 mg GSPE/kg BW or 200 mg GSPE/kg BW (GSPE 25, GSPE 100, and GSPE 200, n = 10 per group) for 12 weeks. (**a**) Representative light microscopy images of retroperitoneal white adipose tissue (rWAT) stained with hematoxylin and eosin from each group; (**b**) Adipocyte area measured from the representative light microscopy images from each group; (**c**) Adipocyte volume calculated from the adipocyte area; (**d**) Total adipocyte number extrapolated from the size of adipocytes and the weight of rWAT; (**e**) Frequency of adipocyte size calculated from the adipocyte area. The values are the means ± SEM of five fields per animal from three animals of each group. Different letters indicate significant differences between groups using one-way ANOVA. Significance was considered when *p* ≤ 0.05.

Histological images of the rWAT in the CAF diet-fed rats showed an increase in the size of adipocytes compared to those in the STD diet-fed rats. Nevertheless, the histological images of rWAT in the CAF diet-fed rats supplemented with GSPE were more similar to those in the STD diet-fed rats (Figure 1a shows representative histological images from the four groups of animals).

A more detailed analysis of these images showed that the adipocyte area and volume of the rWAT were significantly higher when the animals were fed a CAF diet than the STD diet, even though the adipocyte number was not affected by the diet. This was further supported by the observation of the increase in the frequency of adipocytes with areas greater than 2800 µm^2^ in the CAF diet-fed animals (Figure 1b–e). Additionally, the daily administration of GSPE together with the CAF diet prevented the increase in the area and volume of the rWAT adipocytes in a dose-dependent manner, resulting in a lower frequency of adipocytes with areas greater than 2800 µm^2^ also in a dose-dependent manner, eventually reaching similar levels as those in the STD diet-fed rats at 200 mg GSPE/kg BW. Interestingly, the adipocyte number was increased when the rats were fed a CAF diet with the daily administration of GSPE for 12 weeks compared to the CAF diet-fed rats without GSPE. Particularly, at the dose of 200 mg GSPE/kg BW, the number of adipocytes showed an 82% increase compared with that observed with the CAF diet-fed rats without GSPE. Thus, when the rats were fed a CAF diet and daily supplemented with GSPE, the rWAT expansion was modified to increase hyperplasia and reduce hypertrophy compared to the rats without GSPE where only the adipocyte size increased, but not the number.

Finally, we performed a correlation analysis of the adipocyte volume and number in rWAT with the BW, WAT weights, and plasma parameters to test if the morphological changes in the rWAT correlated with the improvement of dyslipidemia by GSPE. The results showed that the adipocyte volume was positively correlated with BW and eWAT weight, and the adipocyte number was negatively correlated with the TC/high-density lipoprotein cholesterol (HDL-c) ratio (Table S2).

### 2.3. Daily Administration of GSPE with a CAF Diet Transcriptionally Increases the Differentiation of rWAT Adipocytes

**Figure 2:**
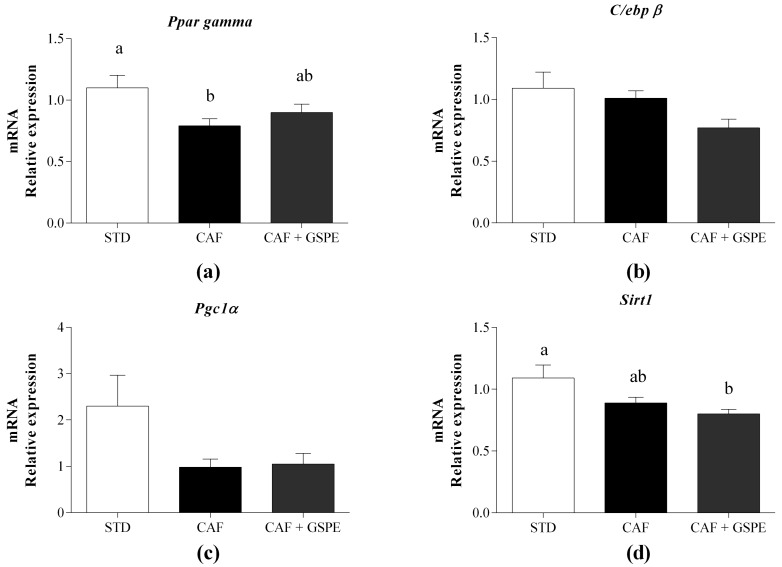

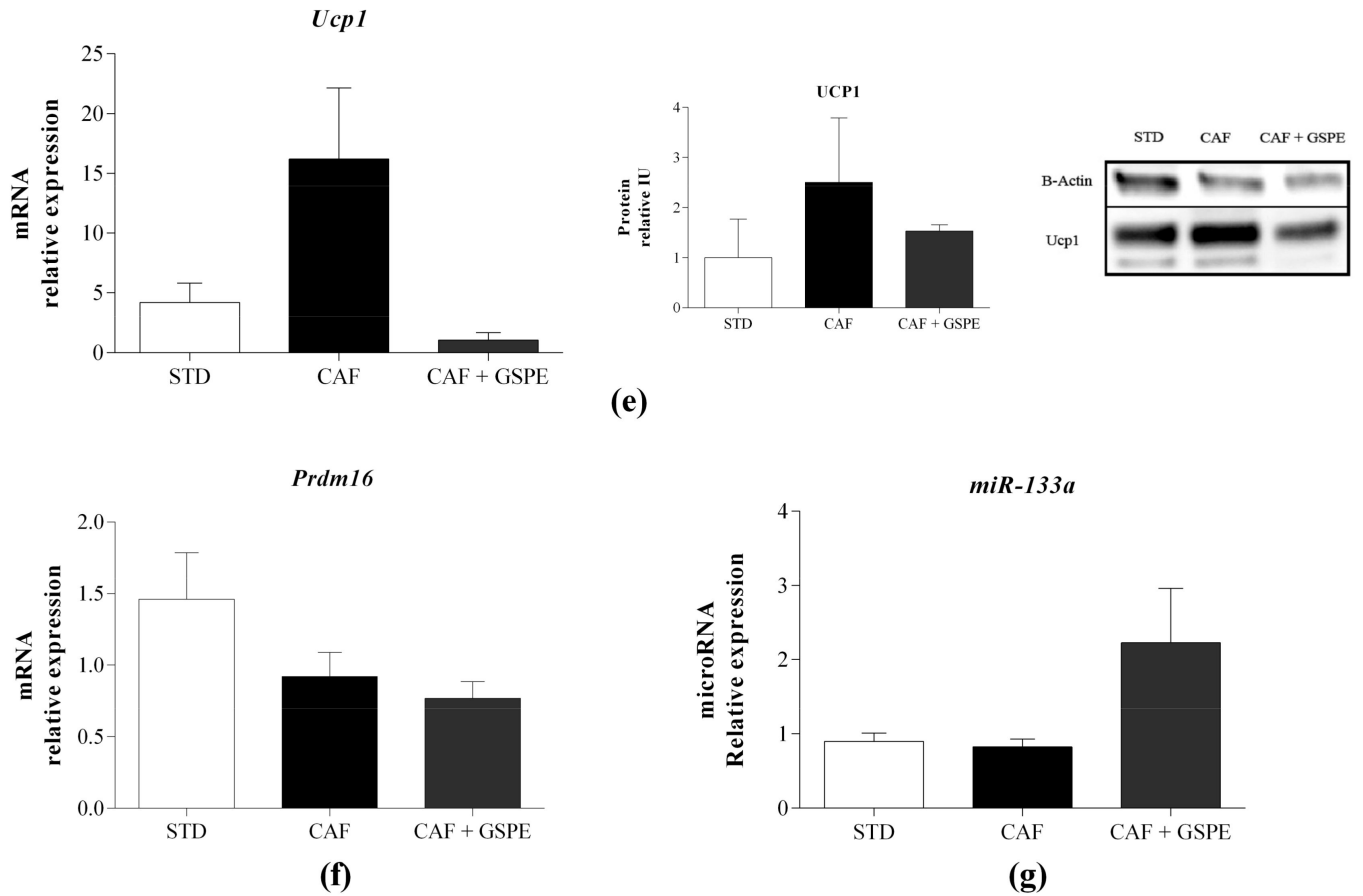
Effect of the long-term administration of a grape seed proanthocyanidin extract (GSPE) with an obesogenic diet on the expression of genes related to adipogenesis and browning in retroperitoneal white adipose tissue. Rats were fed either a standard chow diet (STD group, n = 10), a cafeteria diet (CAF group n = 10), or a cafeteria diet with daily administration of 200 mg GSPE/kg BW (CAF + GSPE, n = 10) for 12 weeks. (**a**) Level of peroxisome proliferator activated receptor gamma (Pparγ) mRNA; (**b**) Level of CCAAT/enhancer binding protein (C/EBP) beta (C/ebpβ) mRNA; (**c**) Level of Pparγ coactivator 1 alpha (Pgc1α) mRNA; (**d**) Level of Sirtuin 1 (Sirt1) mRNA; (**e**) mRNA and protein levels of uncoupling protein 1 (UCP1); (**f**) Level of PR domain containing 16 (Prdm16) mRNA; (**g**) microRNA-133a levels. The values are the means ± SEM. Statistical analyses were performed using the *t*-test. Different letters indicate significant differences between groups when *p* ≤ 0.05.

We also evaluated, at the gene expression level, whether the GSPE administration with the CAF diet can regulate adipocyte differentiation and energy homeostasis. For this, we quantified the mRNA levels of Pparγ, CCAAT/enhancer binding protein (C/EBP) beta (C/ebpβ), and Sirtuin 1 (Sirt1) at the dose of 200 mg GSPE/kg BW, since that was the dose that mostly increased hyperplasia and decreased hypertrophy. The nuclear receptor Pparγ, which is the master regulator of adipogenesis, was upregulated in the CAF diet-fed rats that were daily supplemented with 200 mg GSPE/kg BW compared with the CAF diet-fed rats without GSPE (Figure 2a). In contrast, C/ebpβ, which transactivates Pparγ during adipocyte differentiation [14], was not affected by GSPE (Figure 2b). Similarly, the mRNA level of Pgc1α, which coactivates Pparγ [15], was not altered by the administration of GSPE (Figure 2c). In concordance with the Pparγ upregulation by GSPE, Sirt1, which has been reported to repress Pparγ [16], was repressed by the administration of GSPE (Figure 2d).

As the administration of the highest dose of GSPE in the CAF diet-fed rats attenuated the increase in BW, we also evaluated the browning process of rWAT as a mechanism of the maintenance of the BW during the regulation of the energy balance by proanthocyanidins. The results showed that the daily administration of 200 mg GSPE/kg BW induced a substantial decrease on the level of Ucp1 mRNA compared to the CAF diet-fed rats without the proanthocyanidin administration. This decrease could also be appreciated at the protein level (Figure 2e), even though the differences were not statistically significant, which was likely due to the high degree of variability among the animals. Moreover, the administration of GSPE decreased the expression of Prdm16 and increased the level of microRNA-133a (miR-133a), although the differences were not statistically significant (Figure 2f,g, respectively).

### 2.4. Daily Administration of GSPE with a CAF Diet Affects the rWAT Functionality at the Molecular Level

**Figure 3:**
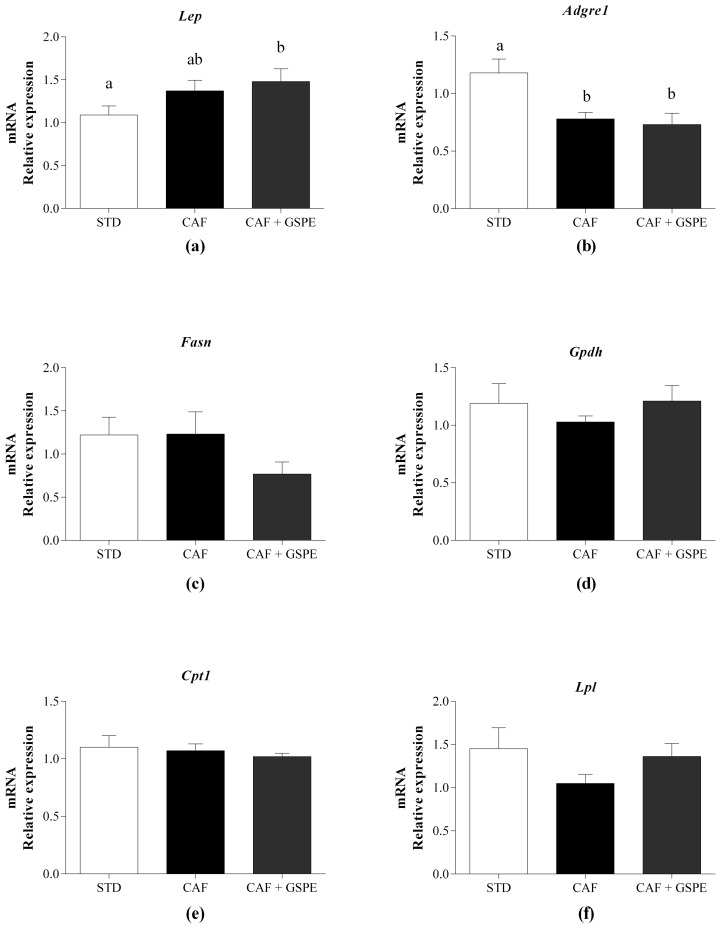
Effect of the long-term administration of a grape seed proanthocyanidin extract (GSPE) with an obesogenic diet on the expression of genes related to adipocyte function in retroperitoneal white adipose. Rats were fed either a standard chow diet (STD group, n = 10), a cafeteria diet (CAF group, n = 10), or a CAF diet with daily administration of 200 mg GSPE/kg BW (CAF + GSPE group, n = 10) for 12 weeks. (**a**) Level of leptin (Lep) mRNA; (**b**) Level of the macrophage surface marker gene adhesion G protein-coupled receptor E1 (Adgre1) mRNA; (**c**) Level of fatty acid synthase (Fasn) mRNA; (**d**) Level of glycerol-3-phosphate dehydrogenase (Gpdh) mRNA; (**e**) Level of carnitine palmitoyltransferase 1A (Cpt1) mRNA; (**f**) Level of lipoprotein lipase (Lpl) mRNA. The values are the means ± SEM. Statistical analyses were performed using the *t*-test. Different letters indicate significant differences between groups when *p* ≤ 0.05.

Aside from the lipogenic modulation of rWAT by GSPE by the regulation of Pparγ, we further evaluated the functional capacity of the rWAT after 12 weeks’ administration of 200 mg GSPE/kg BW with the CAF diet. Since dysfunctional WAT is characterized by leptin resistance and immune cells infiltration, among other factors, we analyzed the expression of leptin in the rWAT, and found that it was slightly increased in the CAF diet-fed animals supplemented with GSPE compared with the animals without GSPE (Figure 3a). Moreover, we demonstrated that the mRNA level of adhesion G protein-coupled receptor E1 (Adgre1), which is a marker of macrophage infiltration that is indicative of dysfunctional rWAT, was not altered by the administration of GSPE (Figure 3b).

We also studied some of the most important genes involved in lipid metabolism in WAT. Regarding the lipogenic markers, we studied the expression of fatty acid synthase (Fasn), which is the principal enzyme involved the de novo synthesis of lipids, and glycerol-3-phosphate dehydrogenase (Gpdh). Fasn was slightly downregulated in the CAF diet-fed animals supplemented with GSPE, although the reduction of the mRNA level of Fasn was not statistically significant (Figure 3c). However, the level of Gpdh mRNA was not modified by GSPE (Figure 3d). Similarly, other genes related to lipid metabolism in the rWAT such as Cpt1, which is the rate-limiting enzyme for fatty acid β-oxidation, or lipoprotein lipase (Lpl), which is the principal enzyme that hydrolyzes circulating TGs into free fatty acids and facilities their entry into adipocytes, were not altered with the administration of GSPE (Figure 3e,f, respectively). In fact, the expression of Fasn and Cpt1 in rWAT was not altered by the cafeteria diet; this could be the precise moment at which the study was realized.

## 3. Discussion

Our research group had previously evaluated the progression of blood pressure, BW, waist parameters, and plasma lipid levels in rats during 12 weeks of CAF diet together with the daily administration of different doses of GSPE (25 mg/kg BW, 100 mg/kg BW, and 200 mg/kg BW) [13]. The results demonstrated that the administration of GSPE with the CAF diet mitigated the obesogenic diet-induced hypertension and dyslipidemia in rats without a clear dose effect. However, only with the dose of 200 mg GSPE/kg BW was a slight protection against the CAF diet-induced BW gain observed [13].

In this study, we used the same cohort of animals to study the effect of the CAF diet with or without GSPE at the end of the feeding period (after 12 weeks of dietary treatment). Our aim was to study whether the attenuation in the development of obesity-associated cardiometabolic risk factors by the long-term administration of GSPE together with a CAF diet is due to a healthier expansion of WAT that prevents the dysfunction of WAT, as well as evaluate the regulation of the energy balance, focusing on the browning process of this fat depot.

To this end, we first evaluated the metabolic damages induced in the rats by the 12 weeks of CAF diet and the preventive effect of this damage by the administration of GSPE. The prolonged feeding of the CAF diet to the rats was seen to increase the GLU, leptin, and TG levels, although the daily administration of GSPE only prevented the CAF diet-induced increase in TGs. In fact, the effect of GSPE on hyperglycemia is not clear, and this proanthocyanidin extract did not reverse the hyperleptinemia of obese animals fed a cafeteria diet [17]. The metabolic damage induced by the obesogenic diet can be a consequence of WAT dysfunction, leading to the inability to store the extra lipids from the diet [3]. Interestingly, the proanthocyanidin hypotriglyceridemic protective effect is in concordance with the preventive decrease in adipocyte hypertrophy in rWAT, since visceral adipocyte hypertrophy has been associated with dyslipidemia [18]. Therefore, the better expansion of WAT induced by GSPE could prevent lipid accumulation in other organs such as the liver [19]. In this regard, the chronic consumption of GSPE showed to reduce TG levels in the liver of cafeteria diet-fed obese rats [20]. Thus, the results of this study are agreement with the well-defined hypolipidemic and cardioprotective effect of proanthocyanidins [9,11]. This study examined rWAT as a representative of visceral WAT, because an excess of visceral fat appears to contribute more to the development of cardiovascular diseases than subcutaneous fat [21], and moreover, this tissue mass was slightly reduced after 12 weeks of a CAF-diet with daily administration of 200 mg GSPE/kg BW.

The prevention of the increase in visceral adipocyte size, and therefore the effect on adipocyte hypertrophy in visceral fat depots, was also reported for other polyphenols or polyphenol-rich extracts, such as isorhamnetin glycosides when they were administered with a high-fat diet in mice [22], resveratrol when it was administered with a high-fat and high-sugar diet in rhesus monkeys [23], and a blackcurrant anthocyanin-rich extract when it was administered with a high-fat and high-cholesterol diet in mice (in the epididymal fat pad) [24]. However, to the best of our knowledge, the capacity of polyphenols or their extracts to modulate hyperplasia has not been previously reported.

Taken this together, this study demonstrated that the administration of GSPE with an obesogenic diet affected the rWAT expansion by increasing adipocyte hyperplasia and reducing hypertrophy, which might prevent the increase in plasma TG levels for the healthier expansion of rWAT, as hyperplasia is associated with the prevention of obesity-associated metabolic disorders. The increase in the adipocyte number in rWAT by preadipocyte differentiation with the supplementation of GSPE at the highest dose (200 mg/kg BW per day) is in concordance with the prevention of CAF diet-induced Pparγ downregulation. Pparγ is considered the master regulator of adipogenesis. However, GSPE has been reported to inhibit adipogenesis by the reduction of Pparγ mRNA levels [25]. This inhibition of adipogenesis was also observed with grape seed procyanidin B2 [26]. Nevertheless, these studies were carried out in 3T3-L1 cells, rather than in animals. The differences between the in vitro and in vivo studies regarding adipogenesis may be because of differences in the compounds targeting the adipocytes. In fact, after the administration of GSPE in rats, flavanols are actively conjugated in the small intestine and liver and metabolized in the colon. Furthermore, after 2 h of administration of 250 mg GSPE/kg BW to the rats, flavanols and their metabolites were found to target mesenteric WAT (mWAT) [27]. Thus, the differences in the version of the molecules targeting the fat depots between the in vivo and in vitro studies could, at least in part, explain the differences in the results.

Sirt1, which is an NAD^+^-dependent nuclear deacetylase, has been reported to repress Pparγ and thus adipocyte differentiation. Specifically, a downregulation of Sirt1 in 3T3-L3 cells resulted in an increase in the level of Pparγ without affecting the expression of C/ebp-β [16]. In concordance with the prevention of the CAF diet-induced downregulation of Pparγ, GSPE also promoted the downregulation of Sirt1 induced by the CAF diet. Moreover, the level of C/ebp-β was not affected by GSPE. To the best of our knowledge, the effect of GSPE to Sirt1 mRNA levels has not been studied in adipocytes; however, it was reported to be upregulated in the livers of healthy rats that were supplemented with 5 mg GSPE/kg BW, 25 mg GSPE/kg BW, or 50 mg GSPE/kg BW for 21 days [28], and in the hypothalamus after 13 weeks of administration of 25 mg GSPE/kg BW with a CAF diet [17]. Therefore, the differential regulation of Sirt1 expression by GSPE in rWAT could have resulted from the different experimental conditions, such as the GSPE dose used, or from the different metabolites targeting these different tissues [27].

The CAF diet-induced increase in BW and rWAT was attenuated by the highest GSPE dose without an effect on food intake, as previously reported by Pons et al. [13]. Moreover, Pparγ is also known to be related to the regulation of the browning process [29], which may prevent obesity-related disorders. Therefore, we further studied whether the administration of GSPE with a CAF diet to the rats could regulate the browning of rWAT at the molecular level, thereby regulating the energy balance and BW retention. The remodeling of white-to-brown AT induces the appearance of brown-like adipocytes in the WAT depots, which express UCP1 and increase energy expenditure [30]. This in turn may prevent the occurrence of obesity and related disorders. Ucp1 mRNA and protein levels were not affected by the administration of GSPE, although the high interindividual variability may explain this lack of difference. In fact, other authors have previously shown that the expression of Ucp1 in rWAT is highly variable within groups that were evaluated after dietary treatment [31]. Aside from Ucp1, Prdm16 and Pgc1α are crucial transcriptional regulators of the browning process [32]. Furthermore, Pgc1α, which coactivates Pparγ, is involved in the induction of mitochondrial biogenesis and regulates thermogenesis and oxidative metabolism [33]. Additionally, the expression of Prdm16 is regulated by miR-133 [34]. As proanthocyanidins are known to modulate the expression of microRNAs (miRNAs) [9,35], we also studied whether GSPE can regulate miR-133. However, Prdm16, miR-133, and Pgc1α were not modulated by the administration of GSPE, pointing to the inability of this dietary treatment to induce rWAT browning or increase energy expenditure at the molecular level. Nevertheless, it is important to consider the high interindividual variability in the analysis of expression of the markers of the browning process. Thus, we cannot provide a conclusive statement regarding the effect of GSPE on rWAT browning. Nonetheless, other dietary polyphenols have been reported to induce the browning of WAT. Along those lines, a combination of resveratrol with quercetin induced the browning of rWAT in obesogenic diet-fed rats [36]. In that study, resveratrol alone and the combination of both polyphenols increased the protein expression of UCP1. Additionally, black tea, which contains catechins and theaflavins, has been shown to increase the levels of UCP1 mWAT [37].

It has been suggested that the failure of WAT to store extra energy affects some of the key factors involved in lipogenesis [4]. Pparγ is also a lipogenic regulator, and its activation in WAT is known to increase the storage of extra fatty acids [38]. Moreover, regarding WAT lipogenesis, although GSPE did not affect the expression of Gpdh, it may slightly mitigate the de novo synthesis of lipids, as the CAF diet-induced increase in Fasn expression was slightly diminished, indicating a minor reduction in the lipogenic activity of rWAT, and pointing to the decrease in the TG accumulation of the adipocytes. However, the expression of markers of lipid mobilization and fatty acid β-oxidation in the adipocytes were not affected by the administration of GSPE, as seen by the lack of change in Lpl and Cpt1, respectively. Other polyphenols have been shown to downregulate Fasn in adipocytes [39]. For instance, resveratrol has been reported to reverse the high-fat diet-induced upregulation in FASN in mouse epididymal WAT (eWAT) [40].

Dietary and pharmacological doses of proanthocyanidins prevent visceral aberrant adipocyte morphology when animals are fed with an obesogenic diet, because proanthocyanidins increase adipocyte hyperplasia and reduce adipocyte hypertrophy in a dose-dependent manner. These results suggest that the preventive effect of proanthocyanidins on cardiometabolic risk factors produced by an obesogenic diet could be a consequence of the inhibition of the failure of visceral WATs. Although more studies in humans are necessary, the administration of proanthocyanidins in individuals consuming a high-fat and high-carbohydrate diet might prevent the development of obesity-related pathologies by improving WAT expansion.

## 4. Materials and Methods

### 4.1. Grape Seed Proanthocyanidin Extract

The GSPE was kindly provided by Les Dérives Résiniques et Terpéniques (Dax, France). The composition of the GSPE has been previously characterized by Margalef et al. [41], and is described in Table S3.

### 4.2. Animal Experimental Procedure

In this study, we used the same cohort of animals as the previous study reported by Pons et al. [13] (License number 8867 from Generalitat de Catalunya). Briefly, six-week-old male Wistar rats (Charles River Laboratories, Barcelona, Spain) were randomly divided in five groups. The standard group (STD, n = 10) was fed a standard chow diet (Panlab A04, Panlab, Barcelona, Spain), the cafeteria control group was fed a CAF diet (CAF, n = 10), and three CAF groups were supplemented with three different doses of GSPE: 25 mg/kg BW (GSPE 25, n = 10); 100 mg/kg BW (GSPE 100, n = 10); or 200 mg/kg BW (GSPE 200, n = 10). After 12 weeks, animals were fasted for 6 h and sacrificed. Blood from the saphenous vein was collected using heparin (Deltalab, Barcelona, Spain) as the anticoagulant. Plasma was obtained by centrifugation (1500× *g*, 20 min, 4 °C), and the WATs were excised and immediately frozen in liquid nitrogen. Both plasma and tissues were stored at −80 °C until further use.

The adiposity index was computed as the sum of the weights of mWAT, rWAT, and eWAT depots.

### 4.3. Quantification of Plasma Parameters

The level of insulin and leptin in the plasma was measured by ELISA (ELISA kit EZRMI-13K and ELISA kit EZRL-83K, Millipore Ibérica, Madrid, Spain). Plasma triglycerides (TGs), total cholesterol (TC), high-density lipoprotein cholesterol (HDL-C), and glucose (GLU) were measured with enzymatic colorimetric kits (QCA, Barcelona, Spain). Non-HDL-C was calculated by subtracting HDL-C from the level of TC. Moreover, HDL-C/non-HDL-C and TC/HDL-C ratios were calculated using the individual values of each animal. Homeostasis model assessment-estimated insulin resistance (HOMA-IR) and quantitative insulin sensitivity check index (QUICKI) indexes were calculated from insulin and GLU plasma levels.

### 4.4. Adipose Tissue Morphology

Adipocyte size and number were determined by microscopic analyses in rWAT after histological staining with hematoxylin-eosin. Small pieces of frozen rWAT (−80 °C) were sent to ELDINE Patologia (Tarragona, Spain) where they were thawed and fixed in 4% formaldehyde. After 24 h of fixation, successive dehydration (alcohol/ethanol 70%, 96%, and 100%; plus xylol/dimethylbenzene) and paraffin infiltration and immersion at 52 °C (Citadel 2000. HistoStar, Thermo Scientific, Madrid, Spain) were performed. The paraffin blocks were subsequently cut into successive two μ-thick sections (Microm HM 355S, Thermo Scientific). The sections were deposited on slides (JP Selecta Paraffin Bath) and subjected to automated staining (Varistain Gemini, Shandon, Thermo) [42]. Images of the adipose tissue sections were acquired with AxioVision Zeiss Imaging software (Carl Zeiss Iberia, S.L, Madrid, Spain). Finally, the images taken at ×20 were stored and analyzed with Adiposoft software (CIMA, University of Navarra, Pamplona, Spain) to quantify the adipocyte number and area. Five fields per sample and three samples from each group of animals (STD, CAF, GSPE 25, GSPE 100, and GSPE 200) were measured. The area was calculated from the average cell area in all of the measured fields. The total number of cells in the rWAT fat depots was calculated from the values of fat cell volumes.

Fat cell volume was obtained for each captured field by using the formula [43]:

$$\frac{\pi }{6}\times \left[3\sigma^{2}\times \bar{d}+{\bar{d}}^{5}\right]$$

where d is the mean diameter of 100 measured cells in the field, and σ is the standard deviation of the diameter. Then, the fat cell density was applied (0.92 g/mL) to determine the fat cell weight [44]. Finally, the total number of fat cells in the whole rWAT depot of each animal was determined by dividing the total weight of the fat depot by the mean cell weight from all of the captured fields.

### 4.5. RNA Extraction

Total RNA, including small RNAs, was extracted from the frozen rWATs by using TRIzol^®^ reagent (Ambion, MA, USA) according to the manufacturer’s protocol. To isolate both total RNA and miRNA, isopropanol precipitation was performed overnight at −20 °C instead of the 10 min at room temperature as recommended for the isolation of only mRNA. The quality of total RNA was checked with a NanoDrop 1000 Spectrophotometer (Thermo Scientific, Wilmington, DE, USA).

### 4.6. mRNA Quantification by Real-Time qRT-PCR

Relative mRNA levels of adhesion G protein-coupled receptor E1 (Adgre1), CCAAT/enhancer binding protein (C/EBP) beta (C/ebpβ), carnitine palmitoyltransferase 1A (Cpt1), fatty acid synthase (Fasn), glycerol-3-phosphate dehydrogenase (Gpdh), leptin (Lep), lipoprotein lipase (Lpl), Ucp1, Pgc1α, Pparγ, Prdm16, and Sirtuin1 (Sirt1) were analyzed by real-time PCR in the rWATs by using cyclophilin (Ppia) as the endogenous control. Total RNA was retrotranscribed using TaqMan Reverse Transcription Reagents kit (Applied Biosystems, Madrid, Spain), and gene expression was evaluated with a Bio-Rad CFX96 Real-Time PCR System (Bio-Rad Laboratories, Barcelona, Spain) using the iTaq™ Universal SYBR^®^ Green Supermix (Bio-Rad Laboratories, Barcelona, Spain) and gene-specific SYBR primers designed for each gene using FastPCR software (Table S4). The results were normalized to Ppia. The amplification was performed according to the temperature steps of 95 °C for 30 s followed by 40 cycles at 95 °C for 5 s and 60 °C for 20 s. The fold change in the level of mRNA was calculated by the 2^−ΔΔ*C*t^ method, where Δ*C*t = *C*t mRNA − *C*t Ppia and ΔΔ*C*t = Δ*C*t treated samples −ΔCt untreated controls.

### 4.7. miRNA Quantification by Real-Time qRT-PCR

A specific TaqMan probe was used for the analysis of microRNA-133a (Assay ID: 002246, Applied Biosystems, Madrid, Spain). U87 small nuclear RNA (Assay ID: 001712, Applied Biosystems, Madrid, Spain) was used as the endogenous control. Single-stranded cDNAs were synthesized by using the TaqMan MicroRNA Reverse Transcription Kit (Applied Biosystems, Madrid, Spain). The reaction was performed at the following temperature cycles: 16 °C for 30 min, 42 °C for 30 min, and 85 °C for 5 min. Quantitative polymerase chain reaction (qPCR) was performed using the TaqMan Universal PCR master mix (Applied Biosystems, Madrid, Spain) The amplification reaction was performed on an ABI Prism 7300 SDS Real-Time PCR system (Applied Biosystems, Madrid, Spain) at 95 °C for 10 min followed by 40 cycles at 95 °C for 15 s and 60 °C for 1 min. Fold change in the miRNA level was calculated according to the equation 2^−ΔΔ*C*t^, where Δ*C*t = *C*t miR-133a − Ct U87 and ΔΔ*C*t = Δ*C*t treated samples −Δ*C*t untreated controls.

### 4.8. Protein Extraction and Western Blotting

Briefly, 200 mg of rWAT were homogenized in 200 μL of RIPA lysis buffer (15 mM of Tris-HCl, 165 mM of NaCl, 0.5% Na-deoxycholate, 1% Triton X-100, and 0.1% SDS) containing protease inhibitor cocktail (1:1000, Sigma-Aldrich, Madrid, Spain) and 1 mM of phenylmethanesulfonyl fluoride (PMSF, Sigma-Aldrich, Madrid, Spain).

Total protein was determined with the BCA kit (Thermo Scientific, Barcelona, Spain). The samples were then prepared for Western blotting with the addition of sample buffer (0.5 M of Tris-HCl (pH 6.8), 10% glycerol, 2% (*w*/*v*) SDS, 5% (*v*/*v*) β-mercaptoethanol, and 0.05% bromophenol blue). The protein samples were boiled for 5 min, and 40 μg of protein was loaded and separated on a 10% SDS-polyacrylamide gel made with the TGX Stain-Free™ FastCast™ Acrylamide Kit (Bio-Rad Laboratories, Barcelona, Spain). The samples were then transferred to polyvinylidene fluoride (PVDF) membranes (Bio-Rad Laboratories, Barcelona, Spain) using a Trans-Blot^®^ Turbo™ Transfer System (Bio-Rad Laboratories, Barcelona, Spain) and blocked with 5% (*w*/*v*) non-fat milk in TTBS (Tris-buffered saline plus 0.5% (*v*/*v*) Tween-20) for 1 h. The membranes were incubated at 4 °C overnight with primary monoclonal antibodies directed against UCP1 (ab23841, Abcam, Cambridge, UK) and β-actin (A2066, Sigma, Madrid, Spain) at 1:1000 dilution in the blocking solution. The membranes were then washed in TTBS (Tris-buffered saline including Tween-20) and incubated with a peroxidase-conjugated monoclonal anti-rabbit secondary antibody (Sigma-Aldrich, A1949, Madrid, Spain) at 1:10,000 dilution for 1.5 h at room temperature. The membranes were then washed in TTBS followed by a single wash in TBS (Tris-buffered saline). Chemiluminescence reaction was carried out using the ECL plus kit (Amersham Biosciences, GE Healthcare, RPN2132, Barcelona, Spain) for protein detection. Images were obtained with the GBOX Chemi XL 1.4 image system (Syngene, Cambridge, UK). Bands were quantified with ImageJ software (NIH, MD, USA). The results were expressed as the relative intensity UCP1/β-actin, where the signal for β-actin was used as the loading control.

### 4.9. Statistical Analyses

The results are reported as the means ± SEM of three animals per group for histological analysis. For all other analyses, 10 animals per group were used. Group means were compared using one-way analysis of variance (ANOVA) with IBM SPSS statistics 20.0 software (SPSS, Inc., Chicago, IL, USA). The comparisons were considered significant at *p* ≤ 0.05. Pearson correlations were calculated using all of the values obtained for all of the animals for each variable. The correlations were judged as significant when the two-tailed *p*-values were ≤0.05.

## Figures and Tables

**Table 1:** Plasma parameters in rats after 12 weeks of feeding a standard (STD) diet, a cafeteria (CAF) diet, or a cafeteria diet with the daily administration of different doses of GSPE (CAF + GSPE).

Plasma Parameters	STD	CAF	CAF + GSPE 25 mg/kg BW	CAF + GSPE 100 mg/kg BW	CAF + GSPE 200 mg/kg BW
GLU (mg/dL)	112.27 ± 3.76 ^a^	137.19 ± 5.92 ^b^	150.07 ± 3.11 ^b^	146.41 ± 8.19 ^b^	139.12 ± 4.84 ^b^
Insulin (ng/mL)	2.23 ± 0.28	3.98 ± 0.78	4.03± 0.58	3.86 ± 0.86	3.91 ± 0.54
HOMA-IR	15.52 ± 1.54	32.49 ± 8.15	32.98 ± 7.19	32.65 ± 7.69	29.06 ± 4.40
QUICKI	0.265 ± 0.003	0.251 ± 0.008	0.246 ± 0.005	0.250 ± 0.006	0.249 ± 0.004
Leptin (ng/mL)	11.49 ± 0.55 ^a^	37.52 ± 3.54 ^b^	40.28 ± 4.61 ^b^	40.07 ± 4.38 ^b^	42.62 ± 6.69 ^b^
TG (mg/dL)	39.50 ± 5.44 ^a^	89.97 ± 19.93 ^b^	64.28 ± 7.96 ^ab^	82.56± 10.65 ^b^	67.27 ± 5.48 ^a,b^
TC (mg/dL)	96.75 ± 4.44	112.03 ± 6.29	98.48 ± 1.91	118.60 ± 7.03	107.62 ± 5.76
HDL-C	39.32 ± 4.45	20.22 ± 2.09	33.92 ± 5.77	33.21 ± 5.47	36.72 ± 5.03
Non-HDL-C	58.11 ± 10.72	83.62 ± 9.50	56.36 ± 7.10	93.22 ± 13.10	57.88 ± 5.24
HDL-C/non-HDL-C	0.85 ± 0.31	0.26 ± 0.05	0.80 ± 0.24	0.42 ± 0.17	0.67 ± 0.12
TC/HDL-C	2.85 ± 0.38	5.30 ± 0.65	2.72 ± 0.60	4.73 ± 0.78	2.69 ± 0.34

The values are the means ± SEM (n = 10). Statistical analyses were performed using one-way ANOVA with Scheffe’s or Dunnett’s T3 post hoc test when necessary. Different letters indicate the significant differences between groups of at least *p* < 0.05. Abbreviations: BW: Body weight; GLU: glucose; HOMA-IR: homeostasis model assessment-estimated insulin resistance, QUICKI: quantitative insulin sensitivity check index; TG: triglycerides; TC: total cholesterol; HDL-c: high-density lipoprotein cholesterol and non-HDL-c: all cholesterol not including the high-density lipoprotein cholesterol.

## Supplementary Materials

The supplementary materials are available online at http://www.mdpi.com/1422-0067/19/9/2632/s1.

## Abbreviations

Adgre1	Adhesion G protein-coupled receptor E1
C/ebpβ	CCAAT/enhancer binding protein beta
Cpt1	carnitine palmitoyltransferase 1A
Fasn	fatty acid synthase
Gpdh	glycerol-3-phosphate dehydrogenase
GSPE	grape seed proanthocyanidin extract
Lpl	Lipoprotein lipase
Pgc1α	Pparγ coactivator 1 alpha
Ppar γ	peroxisome proliferator activated receptor gamma
Prdm16	PR domain containing 16
rWAT	retroperitoneal white adipose tissue
Sirt1	Sirtuin 1
UCP1	Uncoupling protein 1
