# Supplementary material for: Grape Seed Proanthocyanidins Improve White Adipose Tissue Expansion during Diet-Induced Obesity Development in Rats
Source: Int J Mol Sci. 2018 Sep 5;19(9):2632. doi: 10.3390/ijms19092632 (PMC6163825; doi:10.3390/ijms19092632)
Supplement: Supplementary file 1 [file ijms-19-02632-s001.pdf]

Table S1. **Body weights and deposition of white adipose tissue in rats after 12 weeks of feeding a standard (STD) diet, a cafeteria (CAF) diet, or a cafeteria diet with the daily administration of different doses of GSPE (CAF+GSPE).**

	STD	*CAF*	*CAF + GSPE 25 mg/kg BW*	*CAF + GSPE 100 mg/kg BW*	*CAF + GSPE 200 mg/kg BW*
Initial body weight (g)	226.13 ± 3.18	235.50 ± 5.11	238.10 ± 4.26	238.70 ± 5.71	239.56 ± 4.86
Final body weight (g)	406.1 ± 12.5[a]	514.0 ± 15.1[b]	513.5 ± 15.3[b]	501.3 ± 22.6[b]	487.4 ± 24.3[ab]
Body weight increment (g)	169.63 ± 13.67[a]	276.14 ± 13.13[b]	275.40 ± 13.13[b]	262.60 ± 18.26[b]	253.89 ± 23.50[b]
rWAT weight (g)	11.94 ± 2.95[a]	23.70 ± 2.31[b]	26.02 ± 1.60[b]	23.49 ± 2.70[b]	22.71 ± 2.40[ab]
eWAT Weight(g)	7.81 ± 0.90[a]	22.60 ± 2.06[b]	22.22 ± 1.88[b]	21.92 ± 2.91[b]	18.53 ± 1.98[b]
mWAT Weight(g)	4.39 ± 0.29[a]	10.83 ± 1.74[b]	10.17 ± 1.13[b]	9.68 ± 1.30[b]	10.26 ± 1.37[b]
sWAT Weight(g)	5.35 ± 0.49[a]	14.25 ± 2.41[b]	13.48 ± 1.85[b]	12.20 ± 1.77[ab]	13.14 ± 1.64[b]
Adiposity index	5.64 ±0.74[a]	10.97 ± 0.77[b]	11.31 ± 0.57[b]	10.22 ± 0.95[b]	10.86 ± 0.71[b]

The values are the means ± SEM (n=10). Statistical analyses were performed using one-way ANOVA. Different letters indicate significant differences between groups of at least $p < 0.05$.

Abbreviations: BW: Body weight; rWAT: retroperitoneal white adipose tissue; eWAT: epididymal white adipose tissue; mWAT: mesenteric white adipose tissue; and sWAT: subcutaneous white adipose tissue.

Table S2. Correlation analysis of the different parameters analyzed in the study with the rWAT adipocyte volume and number of rats fed for 12 weeks with a standard (STD), a cafeteria (CAF) diet, or a cafeteria diet with the daily administration of 25 mg GSPE/kg body weight (BW), 100 mg GSPE/kg BW, or 200 mg GSPE/kg BW.

	**Adipocyte volume**		**Adipocyte number ($x10^6$)**	
**Variables**	*Correlation coefficient*	*p value*	*Correlation coefficient*	*p value*
Adipocyte volume			-0.431	0.108
Adipocyte number ($x10^6$)	-0.431	0.108		
Body weight	**0.554**	**0.040**	-0.030	0.918
rWAT weight	0.330	0.270	0.126	0.681
eWAT weight	**0.558**	**0.038**	-0.171	0.559
mWAT weight	0.259	0.392	0.407	0.167
sWAT weight	0.312	0.277	0.402	0.154
Adiposity index	0.473	0.102	0.136	0.657
GLU (mg/dL)	0.312	0.299	-0.258	0.394
Insulin (ng/mL)	0.306	0.287	0.018	0.951
Leptin (ng/mL)	-0.147	0.665	0.270	0.421
HOMA-IR	0.211	0.489	-0.177	0.563
QUICKI	-0.125	0.684	0.047	0.878
TG	0.235	0.461	-0.125	0.700
TC	0.222	0.511	-0.080	0.815
HDL-c	-0.292	0.445	0.349	0.357
Non-HDL-c	0.149	0.751	-0.676	0.095
HDL/non-HDL-c	-0.541	0.210	0.696	0.082
TC/HDL-c	0.463	0.296	**-0.758**	**0.048**

Correlations were calculated using all of the values obtained for all of the animals for each variable. The table shows the correlation coefficient that illustrates the level of correlation between variables between 0 (no correlation) and 1 (maximum correlation) level. Bold numbers indicate significant correlations ($p \leq 0.05$).

Table S3. Main polyphenols of the grape seed proanthocyanidin extract (GSPE).

Compound	Concentration (mg/g)
Gallic acid	31.07 ± 0.08
Protocatechuic acid	1.34 ± 0.02
Vanillic acid	0.77 ± 0.04
Proanthocyanidin dimer B2	33.24 ± 1.39
Proanthocyanidin dimer B1	88.80 ± 3.46
Proanthocyanidin dimer B3	46.09 ± 2.07
Catechin	121.32 ± 3.41
Epicatechin	93.44 ± 4.27
Dimer gallate	8.86 ± 0.14
Epicatechin gallate	21.24 ± 1.08
Epigallocatechin gallate	0.03 ± 0.00
Epigallocatechin	0.27 ± 0.03
Proanthocyanidin trimer	4.90 ± 0.47
Proanthocyanidin tetramer	0.05 ± 0.01

Adapted from Margalef *et al* [39]. The results are expressed as mg of phenolic compound/g of GSPE on a wet basis as the mean ± SD (n=3).

Table S4. Primer sequences used for real time qPCR analysis.

Gene	Primer sequences (5′ - 3′)	Product size (bp)	Gen Bank accession no/reference
Adgre1	CTTTGGCTATGGGCTCCCAGTC<br>GCAAGGAGGGCAGAGTTGATCGTG	165	NM_001007557.1
Cpt1	TAT CGT CGC ACA TTA GAC C<br>CAT CTA TGA CCT CCT GGC A	751	NM_031559.2
C/EBPβ	CCA CGA CTT CCT TTC CGA CC<br>CGT AGT CGG ACG GCT TCT TG	71	NM_024125.4
Fasn	TCC CAG GTC TTG CCG TGC<br>GCG GAT GCC TAG GAT GTG TGC	260	Sawano T. *et al.* [43]
Gpdh	CCC TTC CTC CAG GCT ACT CT<br>GAG CTC GGA AAG GTC ACA CA	138	NM_022215.2
Lep	CAT TTC ACA CAC GCA GTC GG<br>GCA AGC TGG TGA GGA TCT GT	137	NM_013076.3
Lpl	ACT GGT GGG ACA GGA TGT GG<br>CCG TTC TGC ATA CTC AAA GTT AGG	196	Kroupa O. *et al.* [44]
Pgc1α	TGC CCC TGC CAG TCA CAG GA<br>GCT CAG CCG AGG ACA CGA GG	177	Distel E. *et al.* [45]
Pparγ	AGG ATT CAT GAC CAG GGA GTT<br>AGC AAA CTC AAA CTT AGC CTC CAT	79	Dovinová I. *et al.* [46]
Ppia	CTT CGA GCT GTT TGC AGA CAA<br>AAG TCA CCA CCC TGG CAC ATG	138	NM_017101.1
Prdm16	GTT CTG CGT GGA TGC CAA TC<br>TGG CGA GGT TTT GGT CAT CA	89	XM_008764418.1
Sirt1	TTG GCA CCC ATC CTC GAA<br>ACA GAA ACC CCA GCT CCA	217	XM_006223877.1
Ucp1	CGA GCC AAG ATG GTG AGT TCG ACA<br>GTG GTG ATG GTC CCT AAG ACA CCT	200	NM_012682.2

Abbreviations: Adgre1: adhesion G protein-coupled receptor E1; CPT1: carnitine palmitoyl transferase I; C/EBPβ: CCAAT/enhancer binding protein beta; FASN: fatty acid synthase; GPDH: glycerol-3-phosphate dehydrogenase; LEP: leptin; LPL: lipoprotein lipase; Pgc1α: PPARG coactivator 1 alpha; Pparγ: peroxisome proliferator-activated receptor gamma; Ppia: peptidylpropyl isomerase A (Cyclophilin A); Prdm16: PR domain containing 16; Sirt1: Sirtuin 1; UCP1: uncoupling protein 1.
